# Frequency of *CHEK2 *mutations in a population based, case–control study of breast cancer in young women

**DOI:** 10.1186/bcr933

**Published:** 2004-09-22

**Authors:** Danielle M Friedrichsen, Kathleen E Malone, David R Doody, Janet R Daling, Elaine A Ostrander

**Affiliations:** 1 Divisions of Clinical Research and Human Biology, Fred Hutchinson Cancer Research Center, Seattle, Washington, USA; 2 Public Health Sciences Division, Fred Hutchinson Cancer Research Center, Seattle, Washington, USA; 3 School of Public, Health and Community Medicine, Department of Epidemiology, University of Washington, Seattle, Washington, USA

**Keywords:** breast cancer, case–control study, CHEK2, population based

## Abstract

**Introduction:**

The cell-cycle checkpoint kinase (CHEK)2 protein truncating mutation 1100delC has been associated with increased risk for breast or prostate cancer. Multiple studies have found an elevated frequency of the 1100delC variant in specific stratifications of breast cancer patients with a family history of the disease, including *BRCA1*/*BRCA2 *negative families and families with a history of bilateral disease or male breast cancer. However, the 1100delC mutation has only been investigated in a few population-based studies and none from North America.

**Methods:**

We report here on the frequency of three *CHEK2 *variants that alter protein function – 1100delC, R145W, and I175T – in 506 cases and 459 controls from a population based, case–control study of breast cancer conducted in young women from western Washington.

**Results:**

There was a suggestive enrichment in the 1100delC variant in the cases (1.2%) as compared with the controls (0.4%), but this was based on small numbers of carriers and the differences were not statistically significant. The 1100delC variant was more frequent in cases with a first-degree family history of breast cancer (4.3%; *P *= 0.02) and slightly enriched in cases with a family history of ovarian cancer (4.4%; *P *= 0.09).

**Conclusion:**

The *CHEK2 *variants are rare in the western Washington population and, based on accumulated evidence across studies, are unlikely to be major breast cancer susceptibility genes. Thus, screening for the 1100delC variant may have limited usefulness in breast cancer prevention programs in the USA.

## Introduction

Cell-cycle checkpoint kinase (CHEK)2 has been shown to play a role in cell cycle regulation, apoptosis, and DNA repair, at least in part through phosphorylation of p53 and BRCA1 in response to DNA damage [1,2]. Several studies have reported associations of germline mutations in *CHEK2, *especially the 1100delC mutation, with increased susceptibility to breast and prostate cancer [3-8]. Although *CHEK2 *germline variants other than 1100delC have been associated with prostate cancer risk, these have not yet been shown to be enriched in breast cancer cases [3,4,9,10].

The association between the *CHEK2 *1100delC variant and risk for breast cancer was initially reported by the *CHEK2 *Breast Cancer Consortium [5]. They found that the frequency of the variant was greater among breast cancer patients with a positive family history of breast cancer who do not carry germline mutations in the *BRCA1 *or *BRCA2 *genes, and in families with male breast cancer, as compared with healthy control individuals from the UK, The Netherlands, and North America [5]. Additionally, they noted that the frequency of the 1100delC variant did not differ significantly between breast cancer patients and matched control individuals from a population-based series of young women from the UK (age < 45 years) and of older women from The Netherlands (age ≥ 55 years) [5]. However, neither population-based series included frequency data after stratifying for family history characteristics.

Several additional studies have addressed the association of the 1100delC variant and breast cancer risk in unique populations. In a Finnish study conducted by Vahteristo and coworkers [6], the frequency of the 1100delC mutation was observed to be slightly but not significantly higher in an unselected cohort of breast cancer patients than in control individuals (identified from the Finnish Red Cross Blood Service). Significant enrichment of the variant was found among index cases with a first-degree or second-degree relative with breast or ovarian cancer, and in women with bilateral breast cancer as compared with patients with unilateral disease. Finally, analysis of the variant in a set of patients with positive family history who were not *BRCA1 *or *BRCA2 *germline mutation carriers demonstrated a significantly elevated frequency of the 1100delC variant as compared with controls. These findings from Vahteristo and coworkers [6] and recent work from Oldenburg and colleagues [7] are similar to data from the *CHEK2 *Breast Cancer Consortium, and suggest a significant role played by the 1100delC variant in breast cancer among women with a positive family history of breast cancer whose disease is not attributable to germline mutations in *BRCA1 *or *BRCA2*.

Finally, a study from New York by Offit and coworkers [8] reported a lower frequency of 1100delC carriers in both breast cancer cases and controls as compared with previous studies that largely included Northern European individuals. The 1100delC mutation was identified in 1.0% of cases, which was not statistically different (*P *= 0.10) from that observed among controls (0.3%), who were volunteers from the New York Cancer Project. Compared with the general population frequency in New York, the 1100delC variant appears to be even rarer among breast cancer patients from Spain [11] and India [12], where studies to date have reported no individuals with the 1100delC variant.

To understand better the association of *CHEK2 *variants and breast cancer risk in the general population in the USA, we analyzed the frequency of three *CHEK2 *variants – 1100delC, R145W, and I175T, each of which reportedly alters CHEK2 protein function – in a population based, case–control study of 506 breast cancer cases diagnosed before age 45 years from western Washington state, and a set of 459 frequency matched control individuals.

## Methods

### Study population

A characterization of the study population has previously been reported and is summarized only briefly [13,14]. Cases were identified through the Cancer Surveillance System of Western Washington, a population-based cancer registry and a participant in the National Cancer Institute's Surveillance, Epidemiology, and End Results Program (SEER). Control individuals were identified through random digit dialing and were frequency matched to the cases on 5-year age group and reference year [15]. The study identified all incident first primary breast cancer cases diagnosed before age 45 years, from May 1 1990 to December 31 1992, in women of all races and ethnic backgrounds, who were residents of King, Pierce and Snohomish counties at the time of diagnosis. Information on potential risk factors for breast cancer, including family history, was obtained through a structured in-person interview. The reference date for the interview, a date beyond which exposure information was not collected, was the month and year of diagnosis for cases and a randomly assigned date for controls. Interviews were completed for 642 cases (84.0%) and 608 controls (73.8% overall response rate). Blood was collected from 540 interviewed cases and 476 interviewed controls.

Tested cases tended to be older than untested cases from the study (*P *= 0.001) whereas no such age-related differences were seen in controls. Untested cases were more likely to have advanced stage disease (51.0% of tested and 41.2% of untested cases had local stage disease, 30.2% and 40.4% had regional disease, and 1.4% and 5.9% had distant disease; *P *= 0.001) and were more likely to be deceased at the last follow up in June 2002 (16.6% of tested and 48.8% of untested cases were deceased; *P *< 0.001). For 40% of participants, blood collection was not attempted until after the initial interview, probably accounting in part for these differences. We observed no difference in cases or controls between those tested and untested with regard to family history.

### Molecular methods

Batches of DNA for genotyping were constructed to contain both case and control samples, and genotyping personnel were blinded as to the case–control status of samples. Previously described specific primers for *CHEK2 *exon 10 were used for PCR amplification [16]: 5'-TTA ATT TAA GCA AAA TTA AAT GTC-3' and 5'-GGC ATG GTG GTG TGC ATC-3'. Genomic DNA (25 ng) was amplified using the AccuPrime TAQ DNA polymerase system (Invitrogen, Carlsbad, CA, USA). Touchdown PCR conditions for the 1100delC amplicon were as follows: denaturation at 94°C for 1 min then 94°C for 30 s, 60°C for 30 s, and 68°C for 30 s for seven cycles with the annealing temperature decreasing by 1°C for each cycle, followed by an additional 28 cycles of 94°C for 30 s, 54°C for 30 s, and 68°C for 30 s. The resulting 556 bp amplicon was analyzed by unidirectional DNA sequencing with the reverse primer. The R145W and I175T variants were sequenced from a 409 bp amplicon generated using the following primers: 5'-TTG CCT TCT TAG GCT ATT TTC C-3' and 5'-AAA GGT TCC ATT GCC ACT GT-3'. As above, 25 ng genomic DNA with AccuPrime TAQ DNA polymerase was amplified by touchdown PCR, in which the starting annealing temperature was 64°C and the final annealing temperature was 58°C. For sequencing, the Applied Biosystems Big Dye Terminator Ready Reaction Mix (Foster City, CA, USA) was used in accordance with the manufacturer's recommended protocol.

Genotyping was conducted in 506 cases and 459 controls. Valid results for all participants were obtained for the R145W and I175T variants, whereas results for one case and one control were not obtained for the 1100delC variant.

### Analysis

To assess the relationship between *CHEK2 *variants and breast cancer risk, logistic regression was used to obtain odds ratios as estimates of the relative risk and 95% confidence intervals [17]. All analyses were completed using Stata statistical software (StataCorp LP, College Station, TX, USA).

Because reference age and year were matching variables for the frequency matching employed in the original study, all risk estimates presented are age (continuous)-and reference year (exact)-adjusted.

A subset of the samples analyzed in the study had been screened previously for germline mutations in the breast cancer susceptibility genes *BRCA1 *and *BRCA2 *[18,19]. Cases were selected for *BRCA1*/*BRCA2 *screening on the basis of an age of diagnosis under 35 years and/or a first-degree family history of breast cancer (*n *= 134). In addition, 235 controls were tested for mutations in the *BRCA1 *gene, and 37 of these controls were additionally tested for *BRCA2*. Overall, 110 cases and 33 controls were available for consideration of *CHEK2 *variant frequencies in *BRCA1*/*BRCA2 *mutation negative subjects.

## Results

### Study population characteristics

Tested cases and controls were generally similar with regard to age, menopausal status, and racial distribution (Table 1). Approximately 90% of all participants were Caucasian. Cases more often reported a family history of breast cancer than did controls, particularly a first-degree family history, which was reported by 19.0% of cases and 8.1% of controls.

### Variants and risk for breast cancer

Overall, no statistically significant differences were observed in frequency between cases and controls for any of the three variants tested (Table 2) and all three variants studied were uncommon. For the 1100delC mutation, a 2.9-fold increased risk was observed among cases compared with controls, because six (1.2%) out of 505 cases and two (0.4%) out of 458 controls carried the variant. However, the confidence interval did not exclude 1 and thus chance cannot be excluded as an explanation (95% confidence interval 0.6–14.6).

We examined the frequencies of the *CHEK2 *variants according to age, race, and family history features of the probands (Table 3). All 1100delC deletion carriers were Caucasian. Among the cases, 0.7% (2/280) of those with no family history of breast cancer, none (0/120) with only a second-degree family history, and 4.3% (4/94) of those with a first-degree family history were found to carry the 1100delC variant (*P *= 0.02). Among controls, 0.3% (1/294) of those with no family history, 0.9% (1/115) of those with only a second-degree family history, and none (0/36) of the controls with a first-degree family history carried the 1100delC variant.

One case, or 2.4% of those with a family history of bilateral breast cancer, and one control, or 3.2% of those with a similar family history, were carriers. Cases with a positive first-degree or second-degree family history of ovarian cancer carried an 1100delC variant more frequently (4.4% [2/45]) than did cases with no such family history (0.9% [4/461]; *P *= 0.09). No controls (0/27) with such family history were carriers. Furthermore, 9.1% (2/22) of the cases with a positive family history of both breast and ovarian cancer were found to carry the 1100delC variant (*P *= 0.07). In the overall dataset, only three cases and two controls reported a family history of male breast cancer, and none carried the 1100delC variant.

The R145W variant was rare in this data set, with only one case and no controls carrying the variant (Table 2). The carrier case was diagnosed before age 30 years, was Caucasian, and reported one first-degree relative with breast cancer who was diagnosed after age 45 years and no family history of ovarian cancer, bilateral breast cancer, or male breast cancer (data not shown).

The I175T change was observed in two (0.4%) of 506 cases and four (0.9%) of 459 controls (odds ratio 0.5, 95% confidence interval 0.1–2.6; Table 2). One control carrier was non-Caucasian. One case (0.4%) and three controls (1.0%) with no family history of breast cancer carried the I175T change. The other case carrying this variant was in the group with a first-degree family history of breast cancer (1/94 [1.1%]) and the other control carrying this variant was among controls with a second-degree family history (1/115 [0.9%]). None of the I175T carriers had a family history of ovarian cancer, bilateral breast cancer, or male breast cancer.

### Non-*BRCA1*/*BRCA2 *carriers and *CHEK2 *mutations

Because some studies suggest that the *CHEK2 *1100delC variant acts as a breast cancer modifier in non-*BRCA1*/*BRCA2 *families only [5-7], we considered the subset of women known not to be *BRCA1 *or *BRCA2 *germline mutation carriers. Within this subset of 110 cases and 33 controls, four (3.6%) cases and no (0%) controls carried the 1100delC variant (*P *= 0.27), one case and no controls carried the R145W change, and one case and no controls carried the I175T change. No *CHEK2 *variants were observed in any known *BRCA1 *or *BRCA2 *mutation carrier.

## Discussion

We analyzed three *CHEK2 *variants that are known to disrupt protein function (1100delC, R145W, and I175T) in a population based, case–control study of breast cancer among young North American women. The 1100delC variant is a protein truncating mutation that abrogates CHEK2 kinase activity [20]. R145W has been shown to have disrupted kinase activity [20,21] and I175T is deficient in binding and phosphorylation of Cdc25A and in binding to BRCA1 and p53 [20-22]. Although an enrichment in the 1100delC variant and a reduction in I175T carriers in the cases were noted, no statistically significant association between any of the *CHEK2 *variants and breast cancer risk was observed. The absolute number of participants carrying *CHEK2 *variants was relatively small, and thus there was limited power to examine frequencies according to family history features. Nonetheless, among cases there was some suggestion that the 1100delC variant may be slightly more frequent in those with a positive first-degree family history of breast cancer (*P *= 0.02) and in those with any family history of ovarian cancer (*P *= 0.09). However, in agreement with two other breast cancer studies [9,10], we observed no suggestive correlation between the R145W and I175T *CHEK2 *variants and breast cancer risk. No *CHEK2 *variants were seen among women found previously to carry a *BRCA1*/*BRCA2 *mutation.

Our overall frequency results for 1100delC of 1.2% for cases and 0.4% for controls are similar to the frequencies reported previously from the UK, Philadelphia, and New York [5,6,8]. In a population based series of individuals from the UK and The Netherlands, the frequency of the 1100delC variant was higher among cases, but did not differ significantly from a set of matched controls (1.3% and 2.5% for cases and 0.3% and 1.2% for controls, respectively) [5]. Likewise, the frequency of 1100delC in a Finnish series of breast cancer patients was similar to that reported among control individuals from the Finnish Red Cross Blood Transfusion Service (2.0% and 1.4%, respectively; *P *= 0.18) [6]. Finally, in North America the 1100delC variant was identified in 1.6% of index cases from breast cancer families in Philadelphia and in 0.6% of control individuals (from the same neighborhood or spouses marrying into a breast cancer family from the same area) [5]. In New York examination of the 1100delC variant in 192 women with a family history of breast cancer, 92 women with a personal history of breast cancer, and 16 male breast cancer patients [8] revealed a mutation frequency of 1.0%, which did not differ significantly from the frequency of 0.3% found in volunteers for the New York Cancer Project (*P *= 0.10).

Several previously published studies [5-7,23] reported an elevated frequency of the *CHEK2 *1100delC variant in specific stratifications of breast cancer patients. Specifically, individuals with positive family history (especially those who are *BRCA1*/*BRCA2 *mutation negative), patients with bilateral disease, and patients with a family history of male breast cancer had a higher occurrence of 1100delC variants as compared with control individuals. We found no *CHEK2 *variants in women with a family history of male breast cancer, but there were only five individuals with such a history in our entire sample. This finding is similar to those of other recent studies that did not find an association between 1100delC and risk for male breast cancer [24-26]. Although the frequency of 1100delC carriers was higher in cases (2.4%) and controls (3.2%) with a family history of bilateral disease as compared with cases (0.7%) and controls (0.3%) with no family history and cases (1.8%) and controls (0) with only a family history of unilateral disease, this was based on sparse data and family history of bilaterality contributed no insights beyond family history overall.

After stratifying by family history, we did observe an elevated frequency of 1100delC carriers among cases with a first-degree family history (4.4%; *P *= 0.02). Although our numbers are small, this frequency is similar to the frequencies reported by others. Vahteristo and coworkers [6] reported that, among 1035 breast cancer patients, 3.1% of those with at least one affected first-degree or second-degree relative were 1100delC carriers. Additionally, in index cases with a family history of breast cancer, Meijers-Heijboer and coworkers [5] observed that 3.0% (31/1036) were 1100delC carriers.

Thus far, the most convincing evidence for an association between the 1100delC variant and breast cancer risk is in families who do not carry *BRCA1*/*BRCA2 *germline mutations [5-7]. However, the 1100delC frequency in *BRCA1*/*BRCA2 *mutation positive families did not differ significantly from the frequency observed among controls [5,6]. In this study we observed that four of 110 cases (3.6%) and none of 33 controls who were known to be *BRCA1 *or *BRCA2 *negative carried the *CHEK2 *1100delC variant. The number of women in the present study with a first-degree family history of breast cancer who tested negative for *BRCA1*/*BRCA2 *mutations (71 cases, 27 controls) does not offer adequate power to detect differences in the frequency of *CHEK2 *variants within this stratification.

The significance of the *CHEK2 *1100delC mutation in individuals with a family history of ovarian cancer is not as well understood. Vahteristo and coworkers [6] found no association between the 1100delC variant and ovarian cancer family history among women with familial breast cancer (0/40). However, Meijers-Heijboer and colleagues [5] reported that 4.0% of index cases or 4.3% of all cases with at least one family member with ovarian cancer carried the 1100delC variant (*P *= 0.016). This is compatible with the frequency we observed (2/45 [4.4%]) among breast cancer cases with a family history of ovarian cancer. Although the numbers are small, our data suggests that further investigation into the association between the *CHEK2 *1100delC mutation and ovarian cancer risk is warranted.

The results of our study should be assessed with regard to its limits. Specifically, there are differences between tested and untested women. Tested cases were more likely to be alive, older, and have a less advanced stage of cancer than untested cases. Thus, the generalizability of these results, although from a population-based study, must be viewed within that context. As noted earlier, the literature is diverse in terms of its estimates of *CHEK2 *mutation frequency. Although the overall sample size of our study was generous (965 women), the frequency of the *CHEK2 *variants turned out to be quite low. As a result, the study had somewhat reduced power, particularly for assessing mutation frequency according to various family history characteristics.

## Conclusion

The population based, case–control study of young women (age at diagnosis < 45 years) presented here does not identify any of the 1100delC, R145W, and I175T variants as major factors in breast cancer susceptibility in western Washington. After stratification by family history characteristics, an association with first-degree family history of breast cancer and possibly family history of ovarian cancer was observed. However, no particular relationship was found with family history of bilateriality or family history of male breast cancer. These results suggest that incorporation of any *CHEK2 *variants into a breast cancer screening program among Caucasian women in the US would be premature. Additional studies, particularly of women with a family history of breast cancer who do not carry mutations in the *BRCA1 *or *BRCA2 *genes, are warranted.

## Competing interests

None declared.

## Abbreviations

bp = base pairs; CHEK = cell-cycle checkpoint kinase; PCR = polymerase chain reaction.

## Figures and Tables

**Table 1:** Characteristics of cases and controls

Characteristic	Cases (*n *= 506)		Controls (*n *= 459)	
	
	*n*	%	*n*	%
Age at reference (years)				
< 35	62	12.3	80	17.4
35+	444	87.7	379	82.6
Race				
White	450	88.9	412	89.8
Nonwhite	56	11.1	47	10.2
Family history				
None	280	56.7	294	66.1
First degree	94	19.0	36	8.1
Second degree	120	24.3	115	25.8
Unknown	12		14	
Menopausal status				
Premenopausal	445	88.3	392	85.8
Postmenopausal	59	11.7	65	14.2
Unknown	2		2	

**Table 2:** Association of *CHEK2 *variants with breast cancer risk

*CHEK2 *status^a^	Cases (*n *= 506)		Controls (*n *= 459)		OR^b^	95% CI
			
	*n*	%	*n*	%		
1100del C						
Noncarrier	499	98.8	456	99.6	1.0	Reference
Carrier	6	1.2	2	0.4	2.9	(0.6–14.6)
R145W						
Noncarrier	505	99.8	459	100.0	1.0	Reference
Carrier	1	0.2	0	-	-	-
I175T						
Noncarrier	504	99.6	455	99.1	1.0	Reference
Carrier	2	0.4	4	0.9	0.5	(0.1–2.6)

^a^All carriers are heterozygous. ^b^Adjusted for age at reference and reference year. CI, confidence interval; OR, odds ratio.

**Table 3:** Frequency of *CHEK2 *variants in cases and controls according to age and family history features

**Characteristic**	All women		1100delC+						I175T+					
	
	Cases	Controls	Cases			Controls			Cases			Controls		
	
	*n*	*n*	*n*	%	95% CI	n	%	95% CI	*n*	%	95% CI	*n*	%	95% CI
Age (years)														
< 30	10	16	0			0			0			0		
30–34	52	64	0			0			0			1	1.6	0.04–8.4
35–39	156	140	1	0.6	0.02–3.5	1	0.7	0.02–3.9	1	0.6	0.02–3.5	0		
40–44	288	239	5	1.7	0.6–4.0	1	0.4	0.01–2.3	1	0.3	0.009–1.9	3	1.3	0.3–3.6
Family History of breast cancer^a^														
None	280	294	2	0.7	0.09–2.6	1	0.3	0.009–1.9	1	0.4	0.009–2.0	3	1.0	0.2–3.0
First-degree	94	36	4	4.3	1.2–10.5	0			1	1.1	0.03–5.8	0		
Second-degree only	120	115	0			1	0.9	0.02–4.7	0			1	0.9	0.02–4.7
Number of relatives with breast cancer^a^														
None	280	294	2	0.7	0.09–2.6	1	0.3	0.009–1.9	1	0.4	0.009–2.0	3	1.0	0.2–3.0
1	137	114	3	2.2	0.5–6.3	1	0.9	0.02–4.8	1	0.7	0.02–4.0	0		
2	51	29	1	2.0	0.05–10.4	0			0			1	3.4	0.09–17.8
3+	26	8	0			0			0			0		
Family history of ovarian cancer														
None	461	432	4	0.9	0.2–2.2	2	0.5	0.06–1.7	2	0.4	0.05–1.6	4	0.9	0.3–2.4
1st or 2nd degree	45	27	2	4.4	0.5–15.1	0			0			0		
Family history of breast and/or ovarian cancer^a^														
No breast/no ovarian	258	278	2	0.8	0.09–2.8	1	0.4	0.009–2.0	1	0.4	0.01–2.1	3	1.1	0.2–3.1
No breast/yes ovarian	22	16	0			0			0			0		
Yes breast/no ovarian	192	141	2	1.0	0.1–3.7	1	0.7	0.02–3.9	1	0.5	0.01–2.9	1	0.7	0.02–3.9
Yes breast/yes ovarian	22	10	2	9.1	1.1–29.2	0			0			0		

^a^Twelve cases and 14 controls had missing information on their family history and are excluded. There were no *CHEK2 *variants in these women.

## References

[B1] Shieh SY, Ahn J, Tamai K, Taya Y, Prives C (2000). The human homologs of checkpoint kinases Chk1 and Cds1 (Chk2) phosphorylate p53 at multiple DNA damage-inducible sites. Genes Dev.

[B2] Bartek J, Falck J, Lukas J (2001). CHK2 kinase: a busy messenger. Nat Rev Mol Cell Biol.

[B3] Seppala EH, Ikonen T, Mononen N, Autio V, Rokman A, Matikainen MP, Tammela TL, Schleutker J (2003). CHEK2 variants associate with hereditary prostate cancer. Br J Cancer.

[B4] Dong X, Wang L, Taniguchi K, Wang X, Cunningham JM, McDonnell SK, Qian C, Marks AF, Slager SL, Peterson BJ (2003). Mutations in CHEK2 associated with prostate cancer risk. Am J Hum Genet.

[B5] Meijers-Heijboer H, van den Ouweland A, Klijn J, Wasielewski M, de Snoo A, Oldenburg R, Hollestelle A, Houben M (2002). Low-penetrance susceptibility to breast cancer due to CHEK2(*)1100delC in noncarriers of BRCA1 or BRCA2 mutations. Nat Genet.

[B6] Vahteristo P, Bartkova J, Eerola H, Syrjakoski K, Ojala S, Kilpivaara O, Tamminen A, Kononen J, Aittomaki K, Heikkila P (2002). A CHEK2 genetic variant contributing to a substantial fraction of familial breast cancer. Am J Hum Genet.

[B7] Oldenburg RA, Kroeze-Jansema K, Kraan J, Morreau H, Klijn JG, Hoogerbrugge N, Ligtenberg MJ, van Asperen CJ, Vasen HF, Meijers C (2003). The CHEK2*1100delC variant acts as a breast cancer risk modifier in non-BRCA1/BRCA2 multiple-case families. Cancer Res.

[B8] Offit K, Pierce H, Kirchhoff T, Kolachana P, Rapaport B, Gregersen P, Johnson S, Yossepowitch O, Huang H, Satagopan J (2003). Frequency of CHEK2*1100delC in New York breast cancer cases and controls. BMC Med Genet.

[B9] Allinen M, Huusko P, Mantyniemi S, Launonen V, Winqvist R (2001). Mutation analysis of the CHK2 gene in families with hereditary breast cancer. Br J Cancer.

[B10] Schutte M, Seal S, Barfoot R, Meijers-Heijboer H, Wasielewski M, Evans DG, Eccles D, Meijers C, Lohman F, Klijn J (2003). Variants in CHEK2 other than 1100delC do not make a major contribution to breast cancer susceptibility. Am J Hum Genet.

[B11] Osorio A, Rodriguez-Lopez R, Diez O, de la Hoya M, Ignacio Martinez J, Vega A, Esteban-Cardenosa E, Alonso C, Caldes T, Benitez J (2004). The breast cancer low-penetrance allele 1100delC in the CHEK2 gene is not present in Spanish familial breast cancer population. Int J Cancer.

[B12] Rajkumar T, Soumittra N, Nancy NK, Swaminathan R, Sridevi V, Shanta V (2003). BRCA1, BRCA2 and CHEK2 (1100 del C) germline mutations in hereditary breast and ovarian cancer families in South India. Asian Pac J Cancer Prev.

[B13] Brinton LA, Daling JR, Liff JM, Schoenberg JB, Malone KE, Stanford JL, Coates RJ, Gammon MD, Hanson L, Hoover RN (1995). Oral contraceptives and breast cancer risk among younger women. J Natl Cancer Inst.

[B14] Daling JR, Malone KE, Voigt LF, White E, Weiss NS (1994). Risk of breast cancer among young women: relationship to induced abortion. J Natl Cancer Inst.

[B15] Waksberg J (1978). Sample methods for random digit dialing. J Am Stat Soc.

[B16] Vahteristo P, Tamminen A, Karvinen P, Eerola H, Eklund C, Aaltonen LA, Blomqvist C, Aittomaki K, Nevanlinna H (2001). p53, CHK2, and CHK1 genes in Finnish families with Li-Fraumeni syndrome: further evidence of CHK2 in inherited cancer predisposition. Cancer Res.

[B17] Breslow NE, Day NE (1980). Statistical Methods in Cancer Research The Analysis of Case–control Studies.

[B18] Malone KE, Daling JR, Thompson JD, O'Brien CA, Francisco LV, Ostrander EA (1998). BRCA1 mutations and breast cancer in the general population: analyses in women before age 35 years and in women before age 45 years with first-degree family history. JAMA.

[B19] Malone KE, Daling JR, Neal C, Suter NM, O'Brien C, Cushing-Haugen K, Jonasdottir TJ, Thompson JD, Ostrander EA (2000). Frequency of BRCA1/BRCA2 mutations in a population-based sample of young breast carcinoma cases. Cancer.

[B20] Wu X, Webster SR, Chen J (2001). Characterization of tumor-associated Chk2 mutations. J Biol Chem.

[B21] Li J, Williams BL, Haire LF, Goldberg M, Wilker E, Durocher D, Yaffe MB, Jackson SP, Smerdon SJ (2002). Structural and functional versatility of the FHA domain in DNA-damage signaling by the tumor suppressor kinase Chk2. Mol Cell.

[B22] Falck J, Mailand N, Syljuasen RG, Bartek J, Lukas J (2001). The ATM-Chk2-Cdc25A checkpoint pathway guards against radioresistant DNA synthesis. Nature.

[B23] Broeks A, de Witte L, Nooijen A, Huseinovic A, Klijn JG, van Leeuwen FE, Russell NS, van't Veer LJ (2004). Excess risk for contralateral breast cancer in CHEK2*1100delC germline mutation carriers. Breast Cancer Res Treat.

[B24] Syrjakoski K, Kuukasjarvi T, Auvinen A, Kallioniemi OP (2004). CHEK2 1100delC is not a risk factor for male breast cancer population. Int J Cancer.

[B25] Neuhausen S, Dunning A, Steele L, Yakumo K, Hoffman M, Szabo C, Tee L, Baines C, Pharoah P, Goldgar D, Easton D (2004). Role of CHEK2*1100delC in unselected series of non-BRCA1/2 male breast cancers. Int J Cancer.

[B26] Ohayon T, Gal I, Baruch RG, Szabo C, Friedman E (2004). CHEK2*1100delC and male breast cancer risk in Israel. Int J Cancer.

